# Using a Combination Approach in Imparting Effective Telephone Referral Skills to Emergency Medicine Residents in Qatar: A Pilot Study

**DOI:** 10.2147/AMEP.S236802

**Published:** 2020-03-09

**Authors:** Khalid Bashir, Yousaf Shah

**Affiliations:** 1 College of Medicine, Qatar University, Doha, Qatar; 2 Emergency Department, Hamad General Hospital, Doha, Qatar

**Keywords:** emergency medicine, telephone referral skills, resident education, learner-centered approach

## Abstract

**Background:**

Appropriate and timely feedback is essential in improving communication skills and is endorsed as a core competency of emergency medicine residency (EMR) education by the Accreditation Council for Graduate Medical Education.

**Aim:**

The study aimed to improve the telephone referral skills through a structured feedback session using ISBAR and to gauge residents’ opinions about this approach.

**Methods:**

This was a pilot, educational project that included emergency medicine residents in current training program from October 2018 to March 2019. A senior faculty observed and audiotaped the telephone referral of the residents during a clinical shift. Personalized feedback was provided immediately after the consultation based on the ISBAR tool. The resident’s opinion was transcribed by the faculty.

**Results:**

Seventeen residents agreed to participate, but 14 (3 female and 11 male) were able to complete the study. Three residents, all male, could not complete the study due to busy clinical shifts. Thirteen (out of 14) really liked this method of feedback “eye opener”, “really helped me to reflect” and 10 indicated to self-record some of their future consultations for self-improvement. Thirteen preferred this form of feedback as compared to conventional feedback. One resident disliked this approach as she was extremely nervous during direct observation and audio recording.

**Conclusion:**

A combination approach of direct observations and audio recordings may be a useful pedagogy in teaching effective telephone referral skills to ED residents.

## Introduction

Appropriate communication is an indispensable skill for doctors and the Accreditation Council for Graduate Medical Education considers it a core competency to be imparted during resident education.^1^ Patient safety may be threatened by the failure of effective communication within healthcare teams.^2^ Internal telephone consultations represent a specific type of communication process in the Emergency Department (ED) of hospitals. Through this method, an ED physician (EP) can request other physicians from inpatient teams to assist in patient care in the form of admission, opinion, or assistance in a special procedure while the patient remains under the care of the EP.^3^ The ED consultation process differs from outpatient consultation where a specialist physician undertakes responsibility for patient care on subsequent visits. EP regularly consults specialty physicians for help in patient management. A study concluded that almost one-third of ED patients require consultation from specialist teams.^3^ Effective telephone consultation skills are fundamental to the efficient operations of the ED. Efficient telephone conversations between emergency medicine (EM) resident and specialists may help prevent misunderstandings or other difficulties that could compromise patient care.^3^,^4^ A survey among final year medical students revealed that two-thirds of the participating final year medical students had received no previous training in referral skills and recommended the inclusion of training in referral skills to their curriculum.^5^ Therefore, it is recommended that EM residents should be formally imparted instruction on telephone consultation skills related to inpatient specialties.^5^,^6^

Learner-centred experiential sessions that necessitate the active engagement of resident physicians constitute the most effective approaches in teaching communication skills and can lead to behavioural changes.^7^ A one-to-one experiential session involves direct observation of a learner’s telephone referral experience by an experienced EP, followed by appropriate and timely feedback and discussion. The feedback can be guided by audio or video recordings of the communication encounter. Feedback provided through the use of technical tools offer many advantages over feedback without such media.^7^–^9^ Learners who are able to observe or to listen to their own recorded interactions can understand their strengths and weaknesses more readily than those who only receive only verbal feedback from the faculty. It is often difficult for residents to recall the communication skills they employed or omitted in a conversation and, thus, an audio or video recording can preserve actual details of the exchange. Such evidence allows learners to experience an objective and accurate self-assessment and grants them the opportunity to revisit a particular aspect of their communication skills.^7^ The support of audio-visual media also greatly improves the accuracy and reliability of feedback, leading to fewer disagreements and misunderstandings. A learner’s resistance to learning is, thus, decreased.^7^ Although aspects of nonverbal communication may be missed in audio recordings, these are less intrusive in the real working environment than a video footage, which is also less important for telephone referrals. Several recommendations have been made in studies on how to improve the referral skills, including senior physician’s supervision and dedicated time and use of digital media. A tool that is highly recommended for improving communication skills is called ISBAR (Table 1). ISBAR is an easy-to-remember and user-friendly tool and has shown a decrease in adverse incidents.^10^ ISBAR may improve the referral quality by presenting a clear picture of the patient’s clinical status to the receiving physician. Presently, adhoc oral feedback is provided to the residents on their telephone referral skills based on observation in a busy ED, which has occasionally led to disagreements between the faculty and residents. To the best of our knowledge, there are no published studies focusing on improving telephone referral skills using direct observation by a senior faculty and audio recordings. This study aimed to improve the telephone referral skills through an organised feedback session and to gauge residents’ opinions about this approach.

**Table 1:** Description of “ISBAR”

I	Introduce yourself and patient (name, location, age)
S	Situation, reason for the telephone call (problem, level of concern)
B	Background (history of presentation, relevant past medical history)
A	Assessment and action (what is the working diagnosis, what has been done so far)
R	Response and rationale (treatments ongoing and completed, investigation completed and pending, plan depending on results/clinical condition

## Methodology

This was a pilot, educational project that included EM residents in a training programme from October 2018 to March 2019. The ED is the largest tertiary care teaching hospital in Doha, the capital of Qatar. The study centre hosts a four-year emergency medicine training programme, which is accredited by the international branch of the USA-based Accreditation Council for Graduate Medical Education. The study was considered exempt by the host hospital’s Research Ethics Committee.

We employed a combination approach of teaching telephone referral skills to EM residents during normal clinical shifts in the ED. One senior emergency physician (KB), who is an instructor at several faculty development workshops including giving and receiving effective feedback, directly observed the full telephone referral, while telephone referral consultation was audiotaped. Afterwards, KB and each resident spend approximately 10 mins in a quieter place of the ED for a feedback session. Initially, KB provided verbal feedback on the observed conversation based on ISBAR tool. Subsequently, the residents reflected on the feedback after listening to the audiotape. Finally, the resident was asked whether they would prefer this method of feedback and to explain the reason for the negative feedback if not. KB observed 14 complete telephone consultations with 14 different residents and provided feedback. The comments were transcribed by the study author. The data were entered into a computer using SPSS (Statistical Package for Social Sciences) version 17 for windows. The categorical variables were mentioned as frequencies and percentages while the continuous variables were expressed as means and standard deviation.

## Results

This prospective, mixed-method study involved 17 EM residents at different levels of training (Table 2). Out of 42 residents, 17 (40%) agreed to participate in the study and 14 (82%) were able to complete the task. Further, 3 (17.6%) male residents, who did not accomplish the assignment, cited their busy clinical shift in the ED as the reason for non-completion. There were 11 males (78.6%) and 3 (21.4%) females. A total of 9 (64.2%) residents (8 males and 1 female) “Introduce” themselves and the patient, while 10 (71.4%) residents (9 males and 1 female) explained the “Situation” during their telephone consultation. Overall, 13 (92.8%) residents (10 males and 3 females) described “Background”, whereas 13 (92.8%) residents (10 males & 3 females) clarified “Assessment”; only 7 (50%) residents (5 males and 2 females) mentioned about their “Recommendation”. Finally, 13 out of 14 (92.8%) residents preferred this method of receiving feedback and majority of them asserted their intention to self-record future telephone consultations for self-improvement. Some of the comments included, “it really helped me to reflect”, “eye opener”. Only 1 (7.3%) resident felt that the audio recording and direct observation by KB made her nervous and disliked this method of feedback.

**Table 2:** Summary of the Results of Telephone Referral Skills

Numbers	Variables	Yes (M/F)	No (M/F)
1	I	9 (8M/1F)	5 (3M/2F)
2	S	10 (9M/1F)	4 (2 M/2F)
3	B	13 (10M/3F)	1 (1M)
4	A	13 (10M/3F)	1 (1M)
5	R	7 (5M/2F)	7 (6M/1F)
6	Preference	13 (10M/2F)	1 (1F)

**Abbreviations:** M, male; F, female.

## Discussion

The proposed method of direct observation followed by oral feedback aided by self-evaluation of the audio recording of an actual telephone consultation may be a useful pedagogical approach for improving telephone referral skills among ED residents. Majority of the residents preferred this approach of education for improving telephone referral skills.

Teaching an effective telephone communication skill to ED residents is one of the most important tasks for the faculty. To avoid compromise with the patient’s safety, it is necessary that the receiving physician is given the clinical information in a succinct manner.^6^ Communication failure is a common reason for medical errors.^11^ A well-structured method of communicating with other physicians by using tools such as ISBAR helps in transferring appropriate information in a timely manner. ISBAR has been extensively used in hospitals in Europe and Australia, particularly among nursing education and inter-hospital transfer.^12^ We used ISBAR in our study, and it was well received by the residents. ED is one of the busiest places in most hospitals around the world and poses different challenges in terms of patient management and education of residents. There is a greater possibility of sudden overcrowding, leading to possible errors and delay in management.^13^ In our study, 3 (17.6%) residents could not complete the study due to the sudden demand in patient care. Audio recording of telephone referral skills from one hospital to another has been successfully used in another study^12^; this method was feasible for our study to protect the patients’ and physicians’ identities.

## Limitations

First, busy ED environment may not allow enough time for such an experiential approach. As evidenced in the case of the three residents who were unable to complete the study, it may be difficult for EP to make time for appropriate feedback, listen to the audio recording and accomplish the requisite self-reflection. Second, some individuals may feel shy and anxious about being recorded or directly observed, which may lead to underperformance, as shown in the case on one resident. Third, we only recorded the voice of the EM residents during telephone consultation, and the doctors’ responses while receiving the consultation were not recorded. Fourth, the residents were only asked about their preference for this method of education. Ideally, this question should have been addressed through a focus group to obtain more detailed and useful information. As this was a pilot study, we restricted it to one question because of the limited time available for the EP. Finally, we did consider the long-term retention of this teaching approach; this was a pilot study, and we hope to run a longitudinal study in the future to observe this effect.

## Conclusion

A combination approach of direct observations and audio recordings may be a useful pedagogy in teaching effective telephone referral skills to ED residents.
